# 86 Multiple dysostosis in Hurler’s disease: a report of three cases

**DOI:** 10.1093/rheumatology/keac496.082

**Published:** 2022-10-07

**Authors:** N Boulemani, EPH El Harrouch

**Affiliations:** Skikda Algeria

## Abstract

**Introduction:**

Hurler syndrome is the most severe form of mucopolysaccharidosis type I (MPS I), It is a hereditary, metabolic disorder due to an enzymatic deficiency in alpha-Liduronidase with tissue accumulation of glycosaminoglycans (GAGs) leading gradually to chronic multiple visceral dysfunction.

We report three cases of multiple dysostosis in Hurler's disease.

Case 1:

Mr S.O, 26 years old man, presenting a dysmorphic syndrome associating short stature, facial dysmorphism, dental staining, gingival retractions, recurrent bronchial infections, mitral valve disease and joint deformities with a tunnel syndrome. Alpha L iduronidase dosage was performed confirming the diagnosis of **Hurler's disease**. The radiological assessment showed thickening of the cranial vault with poorly developed paranasal sinuses. Vertebral bodies collapse with oval shape, thoracolumbar kyphosis, hypoplasia of the iliac bones and the superior acetabular region, coxa-valga, with dysplastic femoral heads; cortical thinning of the long bones with a tapered appearance of the distal portions of the radius and ulna, small and deformed carpal bones, thin metacarpal bones, and dysplastic interphalangeal joints.

Case 2:

Patient B.M 17 years old girl, born of a consanguineous marriage, presenting with a malformation syndrome with short stature, facial dysmorphism, umbilical hernia, valvular heart disease, hepatomegaly, joint stiffness and deformity. Standard x-rays revealed macrocephaly with thickening of the cranial vault, flattened mandibular condyles, with hypertrophy of the clinoid processes; pectus excavatum, platyspondyly with thoracolumbar kyphoscoliosis, small and narrowed pelvis, enlarged and obliquus cotyles, coxa-valga with dysplastic femoral heads; long curved bones with metaphyseal and diaphyseal enlargement, short and enlarged proximal and intermediate phalanx and hypoplastic distal phalanx.

Case 3:

Patient D.W, 31-year-old female, born of a consanguineous marriage. The patient’s physical examination shows a dysmorphic syndrome with short stature, facial dysmorphism, corneal opacity, recurrent otorhinolaryngological and bronchial infections, joint deformities with carpal tunnel syndrome. An alpha L iduronidase assay was performed confirming the diagnosis. The radiological assessment revealed macrocephaly, thickening of the cranial vault, an elongated J-shaped sella turcica, a short thorax, short and thick clavicles, widened, oar-shaped ribs tapering at their vertebral insertion, “spur” vertebrae, thoracolumbar kyphoscoliosis, narrowed pelvis with coxa-valga and genu valgum, small and deformed carpal and tarsal bones with cortical thinning of long bones.

**Discussion:**

The term dysostosis multiplex is used to describe skeletal abnormalities seen in MPS I that are often early and prominent. The progressive accumulation of GAGs led to an increase in chondrocyte apoptosis favoring the production of pro-inflammatory cytokines (TNFα, IL-1), chemokines and metalloproteases. This results in a degradation of the cartilaginous matrix, increased by the mechanical stresses, the disturbance of endochondral ossification, particularly in the sites subjected to articular mechanical stresses. That could explain the focal defect of the ossification of certain cartilaginous sites leading to this multiple dysplasia. At the axial level, the vertebrae can be oval shaped and flattened (platyspondyly). In the lower limbs, coxo-femoral abnormalities (coxa valga, splayed or flattened acetabulum), epiphyseal alterations, genu valgum, and hypoplasia of the iliac bones can be found. The metacarpal bones take on a tapered appearance like sucked candy cane and the interphalangeal joints of the hands and feet are dysplastic. Hypoplasia of the odontoid process leads to potentially serious cervical instability. Regular monitoring by MRI is necessary to avoid the risk of spinal cord section. The early introduction of enzyme replacement therapy led to a slower progression of symptoms, with improved growth, joint mobility and physical capacity, and stability over time, especially when associated with an appropriate rehabilitation care.

**Conclusion:**

Even if Hurler syndrome is rare, it is still underdiagnosed, it can give various and varied clinico-radiographic features and potentially severe disabilities. Early diagnosis is essential since enzyme replacement therapy could stop or slow down the evolution to irreversible tissue damage.

